# A mixed methods study to inform and evaluate a longitudinal nurse practitioner/community health worker intervention to address social determinants of health and chronic obstructive pulmonary disease self-management

**DOI:** 10.1186/s12890-022-01863-w

**Published:** 2022-03-01

**Authors:** Lauren Kearney, Renda Soylemez Wiener, Mohsin Dahodwala, Gemmae M. Fix, Jacqueline Hicks, Frederic Little, Jinesa Howard, Alexis Gallardo Foreman, Cornelia Wakeman, Charles O’Donnell, Katia Bulekova, Mari-Lynn Drainoni, Hasmeena Kathuria

**Affiliations:** 1 The Pulmonary Center, Department of Medicine, Boston University School of Medicine, 72 East Concord Street, R304, Boston, MA 02118 USA; 2 Center for Healthcare Organization & Implementation Research, VA Boston Healthcare System, Boston, MA USA; 3 Center for Healthcare Organization & Implementation Research, VA Bedford Healthcare System, Bedford, MA USA; 4 Section of General Internal Medicine, Department of Medicine, Boston University School of Medicine, Boston, MA USA; 5 Department of Health Law Policy & Management, Boston University School of Public Health, Boston, MA USA; 6 Department of Biostatistics, Boston University School of Public Health, Boston, MA USA; 7 Research Computing Services (RCS) Group, Information Services & Technology, Boston University, Boston, MA USA; 8 Section of Infectious Diseases, Department of Medicine, Boston University School of Medicine, Boston, MA USA; 9 Evans Center for Implementation and Improvement Sciences, Boston University, Boston, MA USA

**Keywords:** Chronic obstructive pulmonary disease (COPD) self-management, Social determinants of health (SDOH), Community health worker, Health related quality of life (HRQOL), Hospital readmission

## Abstract

**Background:**

Individuals with low socioeconomic status experience higher prevalence and worse outcomes of chronic obstructive pulmonary disease (COPD). We undertook a quality improvement initiative at our safety net hospital in which a nurse practitioner (NP)/community health worker (CHW) team followed patients with COPD, frequent admissions, and unmet SDOH needs from hospitalization through one month post-discharge. We report our mixed methods approach to inform development and preliminary evaluation of this intervention.

**Methods:**

We first assessed characteristics of patients admitted with COPD in 2018 (n = 1811), performing multivariable logistic regression to identify factors associated with ≥ 2 admissions per year. We then tested a standardized tool to screen for unmet SDOH needs in a convenience sample of 51 frequently hospitalized patients with COPD. From January–July 2019, we pilot tested the NP/CHW intervention with 57 patients, reviewed NP/CHW logs, and conducted qualitative interviews with 16 patient participants to explore impressions of the intervention.

**Results:**

Patients with Medicaid insurance, mental health disorders, cardiac disease, and substance use disorder had increased odds of having ≥ 2 admissions. COPD severity, comorbidities, and unmet SDOH needs made COPD self-management challenging. Seventy-four percent of frequently admitted patients with COPD completing SDOH screening had unmet SDOH needs. Patients perceived that the NP/CHW intervention addressed these barriers by connecting them to resources and providing emotional support.

**Conclusions:**

Many patients with COPD admitted at our safety-net hospital experience unmet SDOH needs that impede COPD self-management. A longitudinal NP/CHW intervention to address unmet SDOH needs following discharge appears feasible and acceptable.

**Supplementary Information:**

The online version contains supplementary material available at 10.1186/s12890-022-01863-w.

## Introduction

Chronic obstructive pulmonary disease (COPD) is a leading cause of death and hospitalization in the U.S [1–4]. The U.S. Centers for Medicare and Medicaid Services instituted a penalty to hospitals with high risk-adjusted, 30-day all-cause unplanned readmissions rates after an index hospitalization for COPD in 2014 [5]. In response, hospitals have sought ways to improve outcomes for patients with COPD [6–8].

Most COPD discharge care bundles focus on medical care of COPD [9]. In 2016 Boston Medical Center (BMC), a large safety-net hospital, implemented the COPD Readmissions Reduction Program, consisting of a pulmonary nurse practitioner (NP) who provides COPD education during hospitalization for patients with COPD and clinical follow-up within 14 days post-discharge. Yet thirty-day all-cause readmissions after hospitalization for COPD remained high, suggesting short-term approaches focusing on medical care during the immediate post-discharge period do not fully address factors contributing to readmission.

Comorbidities and social determinants of health (SDOH), the social circumstances in which people are born, grow, live, work, and age, increase risk of hospitalization for patients with COPD [6–8, 10–12]. We used a mixed-methods approach [13, 14] to inform and evaluate a revised quality improvement (QI) initiative to reduce COPD readmissions. First, we identified factors associated with multiple admissions among BMC patients with COPD. We systematically assessed unmet SDOH needs using a standardized SDOH screening tool among frequently admitted patients with COPD. Based on our findings, BMC piloted an initiative in which NP’s efforts were paired with a community health worker (CHW) who engaged with patients for one month post-discharge to address barriers to COPD self-management. In this study we report findings of our mixed methods analyses to inform and evaluate feasibility and acceptability of this QI initiative to reduce hospitalizations among high-need individuals with COPD admitted to our safety net hospital.

## Methods

### Study design and overview

We utilized a mixed-method study with an explanatory sequential design through which we used qualitative data to provide insight into the characteristics of patients with COPD who are frequently hospitalized and to understand the feasibility of a NP/CHW intervention [13]. Our study took place at Boston Medical Center (BMC), New England’s largest safety net hospital which serves a substantial population of patients from underserved communities (approximately 72% of patients) [15]. The Institutional Review Board at Boston University Medical Campus approved this study.

In light of the failure of BMC’s initial COPD readmissions program to reduce hospitalizations, we convened an advisory stakeholder panel that included a patient with COPD, clinicians (hospitalist, ambulatory primary care provider, and pulmonary NP), a CHW (hired by the hospital who identifies as multiracial, unilingual (English-speaking), and is a long-time Boston resident), and BMC hospital leaders (Director of Strategy Implementation and Director of the COPD Readmission Reduction team) with the goal of developing a more successful program.

The panel convened in January 2019 to review 2018 admissions’ data pulled from BMC’s electronic health record (EHR) and NP experiences with the COPD Readmissions Reduction Program. To confirm NP’s perceptions that patients frequently experienced unmet SDOH needs that were barriers to COPD self-management, we tested a standardized SDOH screening tool (THRIVE) [16] in a convenience sample of 51 patients with COPD and ≥ 2 admissions in the past year. THRIVE asks eight questions about SDOH domains (housing, food, affording medications, transportation, utilities, caregiving, education, employment; Additional file 1). THRIVE also assesses interest in receiving resources to address identified SDOH needs [16].

After reviewing all data, the stakeholder panel concluded that adults with COPD and ≥ 2 hospitalizations per year with ≥ 1 unmet SDOH need were in need of a tailored intervention. We agreed on essential components: a longitudinal intervention bringing together a pulmonary NP to deliver evidence-based COPD care and a CHW to address unmet SDOH needs. Both the patient and the CHW noted that the intervention would need to be implemented for at least one-month post-discharge, noting that it takes at least 30 days to address SDOH needs (e.g. securing non-emergency medical transportation) experienced by our patients.

As recommended by the stakeholder panel, prior to pilot testing the intervention, the NP received training on guideline concordant COPD recommendations [17]. The CHW completed training on administering the THRIVE screening tool with content focused on how to initiate services tailored to unmet SDOH needs and received CHW core competency training through our hospital. Recognizing the high smoking rates in our patient population, both the NP and CHW received certification in tobacco treatment practice through the Association for the Treatment of Tobacco Use and Dependence [18].

From January–July 2019, we pilot tested the longitudinal NP/CHW intervention. To evaluate feasibility and acceptability of the intervention, we reviewed NP/CHW activity logs and qualitatively examined perspectives of patient participants.

### EHR data

We used BMC Clinical Data Warehouse, which consolidates data from the electronic health record, to identify adults (age ≥ 18 years) admitted to the hospital or clinical observation unit between January 1-December 31, 2018 with COPD (n = 1811). Patients were identified with COPD by ICD-10 codes J44.9 and/or COPD (COPD, chronic bronchitis, and/or emphysema) on discharge problem lists. We collected data on [1] demographics; [2] smoking status; [3] comorbid conditions associated with readmissions [6, 7, 12] (i.e., cardiac conditions, substance use disorders (SUD) and mental health disorders (MHD) identified by ICD-10 codes and/or listed on discharge problem lists); and [4] number of admissions. We calculated median number of days hospitalized per year.

We performed multivariable logistic regression to identify factors associated with ≥ 2 admissions. Demographics (age, gender, race, ethnicity, insurance-type), current smoking status, and comorbid illness (cardiac conditions, SUD, MHD) were selected a priori as variables for inclusion in the multivariable models. Statistical analysis was conducted using the Statistical Language R, version 4.0.2, with *p* values ≤ 0.05 considered significant.

### THRIVE screener administration

We identified 70 participants from a list of patients scheduled in NP’s clinic, which largely consists of patients recently discharged from the hospital who carry COPD as a diagnosis on their problem list. We administered the validated THRIVE screening tool for SDOH needs (Additional file 1) to these patients. We additionally assessed perceived support, cigarette use, stress, anxiety, depression, and substance use (alcohol, drug use or misuse) (included in Additional file 1), all factors which may affect COPD self-management.

### NP/CHW intervention

From January-July 2019, we arranged for the NP’s efforts to be complemented by CHW-navigation for 1 month post-discharge. During this period, we enrolled a convenience sample of 57 patients with COPD, who were admitted ≥ 2 times and reported at least 1 unmet SDOH need to the NP/CHW team. Table 1 describes elements of the NP/CHW intervention, based on recommendations from the stakeholder advisory committee.

**Table 1:** Elements of COPD nurse practitioner (NP)/community health worker (CHW) intervention

	COPD readmission reduction NP tasks	CHW tasks
During hospital	Schedules a follow-up visit within 3–14 days of hospital discharge • Presents options for pharmacotherapy based on smoking behaviors and cravings (derived from ATTUD training) Provides tobacco treatment to individuals who smoke cigarettes • education on how to use medications • personal COPD action plan • how to recognize COPD exacerbations • the hospital discharge plan Provides bedside COPD education and personalized COPD action plan that includes:	Connects patients to tailored resources based on unmet SDOH needs Provides tobacco treatment to individuals who smoke cigarettes • Presents a menu of options for pharmacotherapy based on smoking behaviors and cravings (derived from ATTUD training) Arranges follow-up visit with pulmonary NP within 3–14 days of hospital discharge
Post-discharge	Continues to work with pharmacy, durable medical equipment (DME), Visiting Nurse Association, PCP, and specialists to ensure safe discharge Available to address medical questions (both patients and families) and advise when a patient needs to come into clinic, emergency room, or hospital to receive medical care for COPD or comorbid illnesses Provides ongoing tobacco treatment to individuals who smoke cigarettes Provides tailored education and self-management training to ensure that patients understand the plan provided as an inpatient	Explores SDOH-related issues that are barriers to accessing and engaging in COPD care Connects patients to resources to address unmet SDOH needs Explores the patient’s prior experience with COPD treatment and brainstorms strategies to improve adherence Provides navigation to help patients access medical care Provides ongoing tobacco treatment to individuals who smoke cigarettes Works flexible hours, contacting patients on evenings/weekends as needed

The NP/CHW team kept logs tracking the number of patients engaged during hospitalization and post-discharge and recorded tasks completed. We reviewed these logs to evaluate NP/CHW activities and assess feasibility of the study. The feasibility metrics assessed in team logs included: (1) total time spent by the CHW/NP team on each patient; (2) tasks completed for each patient (e.g., tobacco treatment, connecting to specific SDOH resources, COPD clinical management tasks); and (3) ability to engage patient in program (i.e., # of patients completing post-discharge visits).

### Qualitative data

We conducted semi-structured interviews (January–July 2019) with 16 purposely selected patients based on varying unmet SDOH needs and level of engagement with the CHW/NP. Interviews were audio-recorded with participant consent. Participation was voluntary and no monetary compensation was offered.

**Fig. 1:**
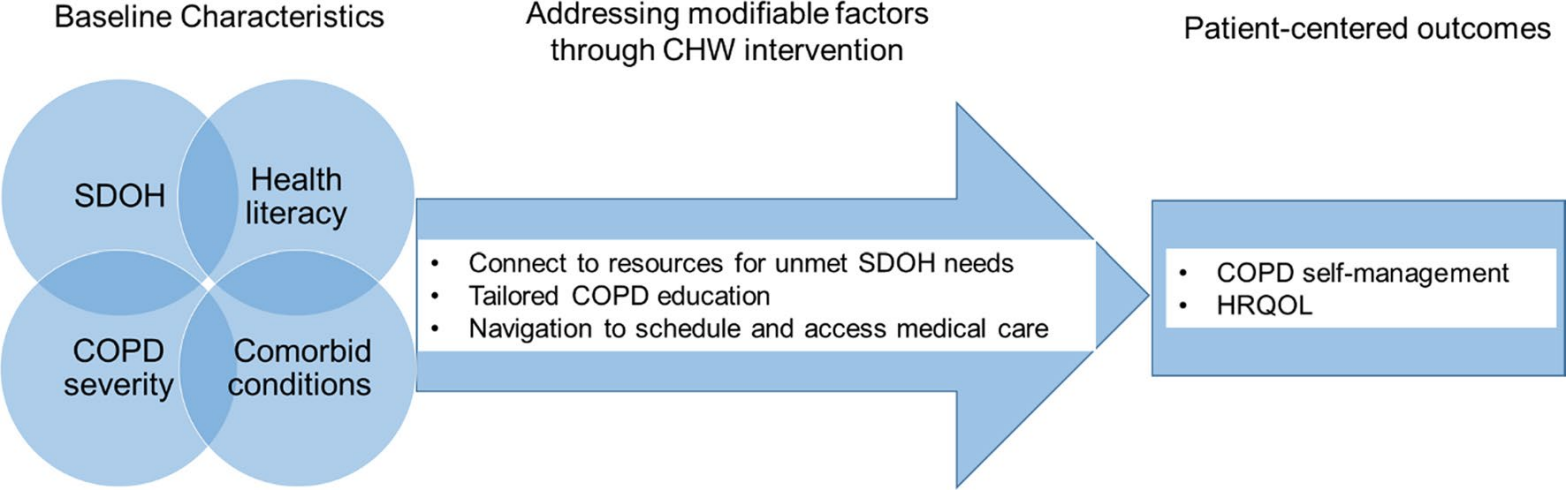
Social-contextual model—adapted for COPD. The Social Contextual Model stresses the influence of social context on health behaviors. The theoretical underpinning of the NP/CHW intervention is to identify modifiable social contextual factors (SDOH such as food insecurity) and to intervene on these modifiable factors (i.e. connect to existing community services to address SDOH needs), thus positively impacting COPD self-management and leading to improved HRQOL

We utilized the Social Contextual Model [19] to develop our interview guide, which provides a framework for categorizing the social context that influences health behaviors (Fig. 1). We probed for how health (COPD severity, co-morbid conditions) and contextual factors (unmet SDOH needs, health literacy) contributes to COPD self-management and HRQOL. The guide was designed to identify modifiable aspects of social context, elicit suggestions on how providers can intervene on these factors, and explore acceptability of the intervention.

We used NVivo to organize and support content analysis. Interviews were transcribed verbatim. Transcripts were analyzed using deductive and inductive analysis [20, 21]. For deductive analysis, data were mapped to constructs from the Social Contextual Model. For inductive analysis, we conducted unstructured coding to allow for new themes. After developing a preliminary codebook, 3 study members independently reviewed all transcripts and revised and added codes until consensus on codes and summary categories were reached. We finalized categories, grouped themes in each category, and identified quotes best highlighting themes.

## Results

### Patient demographics and SDOH needs

Among 1,811 patients admitted with COPD in 2018, 776 (42.8%) had ≥ 2 admissions (median 2), with a median of 12 total days in hospital (IQR 6 to 22 days). Characteristics of those with one admission compared to those with ≥ 2 admissions along with adjusted odds ratios (AOR) with 95% confidence intervals (CI) are reported (Table 2). Medicaid-insured patients had 70% increased odds of ≥ 2 admissions (AOR 1.7, 95% CI 1.1–2.7). Patients with the following co-morbidities had increased odds of ≥ 2 admissions: MHD (AOR 1.5, 95% CI 1.1–1.9), cardiac disease (AOR 2.0, 95% CI 1.5–2.8) and SUD (AOR 1.8, 95% CI 1.4–2.4) (Table 2).

**Table 2:** Factors associated with multiple admissions among all patients admitted with COPD in 2018

		1 admit (n = 1035)	≥ 2 admits (n = 776)	AOR of ≥ 2 admits (95% CI), p-value
Gender	Male	555 (53.6%)	411 (53.0%)	1.0 (.8, 1.2), 0.74
Ethnicity	Non-Hispanic	923 (89.2%)	692 (89.2%)	
	Hispanic	103 (10.0%)	84 (10.8%)	1.4 (0.9, 2.2) 0.09
Race	White	540 (52.2%)	362 (46.6%)	
	Black	333 (32.2%)	316 (40.7%)	1.1 (0.9, 1.4), 0.07
	Other	22 (2.1%)	10 (1.3%)	0.5 (0.2, 1.2), 0.24
	Unknown	140 (13.5%)	88 (11.3%)	0.6 (0.4, 1.0), 0.1
Age	Mean (SD)	66.6 (12.1)	66.7 (12.0)	1.0 (0.995, 1.02), 0.29
Medicaid-insured		354 (34.2%)	359 (46.3%)	**1.7 (1.1–2.7), 0.01**
Currently smokes cigarettes		453 (43.8%)	381 (49.1%)	1.1 (.9, 1.4), 0.47
Substance use disorder*		190 (18.4%)	221 (28.5%)	**1.8 (1.4, 2.4), < 0.01**
Mental health disorders**		249 (24.1%)	201 (25.9%)	**1.5 (1.1, 1.9), 0.01**
Cardiac illness***		836 (80.8%)	695 (89.6%)	**2.0 (1.5, 2.8), < 0.01**

^*^Use disorders includes the following substances: alcohol, opioids, cannabis, cocaine, sedatives, stimulants, hallucinogens, inhalants

^**^Mental health disorders includes mood disorders, schizophrenia, anxiety disorders, psychotic disorders

^***^Cardiac illness includes congestive heart failure, coronary artery disease, and hypertension

Boldface indicates statistical significance

AOR, adjusted odds ratio; CI, confidence interval

Of the 70 patients identified from a list of patients scheduled in NP’s clinic following hospital discharge, 19 (27%) could not be reached. Another nine patients were excluded because they did not meet criteria of ≥ 2 admission per year. Comparison of characteristics (age, gender, race, ethnicity and insurance type) of participants (n = 42) with those not reachable (n = 19) did not differ by Chi-square and student’s *t*-tests. Table 3 shows demographics of the 42 patients with ≥ 2 admissions who completed THRIVE. Seventy-four percent (31/42) screened positive for unmet SDOH needs. Overall, 87.0% (27/31) expressed interest in help with resources and 75% (24/32) expressed interest in meeting a CHW to address needs.

**Table 3:** Self-reported characteristics of patients with ≥ 2 admissions who completed the THRIVE screener

		Total	Percent of patients
*Demographics (n = 42)*			
Gender	Male	24	57.1
Ethnicity	Hispanic	2	4.7
Race	Black	23	54.8
	White	16	38
	Unknown/missing	3	7.1
Age	Mean	57.9	
Insurance	Medicaid (primary or dual-insured)	36	85.7
	Medicare	4	9.5
	Commercial	2	4.7
*Potential barriers to COPD self-management (n = 42)*			
Currently smokes cigarettes		26	61.9
Alcohol use		9	30.9
Illicit drug use		5	11.9
Anxiety		20	47.6
Depression		5	11.9
Social isolation		8	19
Unmet SDOH needs		31	73.8
*Unmet SDOH needs (n = 31)*			

SDOH domain assessed by THRIVE screening		Reported SDOH need, number (%)	Willingness to accept help, number (%)*
Housing insecurity		9 (29%)	6 (66.7%)
Food insecurity		16 (51.6%)	6 (37.5%)
Transportation (trouble getting to medical appointments)		17 (54.8%)	7 (41.2%)
Education (interested in more education)		1 (3.2%)	1 (100%)
Employment (unemployed and looking for a job)		5 (16.1%)	4 (80%)
Medications (trouble paying for medications)		3 (9.7%)	1 (33.3%)
Utilities (trouble paying for heating or electricity)		7 (16.7%)	4 (57.1%)
Caregiving (trouble taking care of child, family or friend)		0 (0%)	N/A

*Number represents willingness to accept help among those with reported SDOH need

### NP/CHW activities

The CHW/NP team met all 57 patients participating in the QI initiative during admission (Table 4). The most common tasks completed during hospitalization included providing COPD education (NP), facilitating scheduling in pulmonary clinics (NP and CHW), arranging transportation for medical appointments (CHW), and providing tobacco treatment (NP and CHW). Review of the NP/CHW tracking logs demonstrated that commonly performed post-discharge tasks included providing tobacco treatment and COPD education, providing transportation to medical appointments, and facilitating scheduling to medical appointments (Table 4). Other post-discharge tasks included arranging food assistance and addressing other unmet SDOH needs and connecting to medical care including MHD/SUD clinics and pulmonary rehabilitation programs (CHW). The NP was able to meet with 36 patients within 14 days of hospital discharge. The NP spent 2–4 h per patient per week and the CHW spent approximately 4 h per patient per week. CHW visits most commonly occurred in the pulmonary clinic or over the phone; a minority of visits occurred in patient’s homes.

**Table 4:** Tasks completed by NP/CHW team

Tasks/services provided	Number of patients assisted while hospitalized (by whom)	Number of patients assisted post-discharge (by whom)
*Tobacco treatment (n = 35 who currently smoke cigarettes)*		
Smoking Cessation counseling	35 (NP and CHW)	27 (NP and CHW)
Facilitate pharmacotherapy (Chantix, NRT)	24 (NP and CHW)	19 (CHW)
*SDOH concrete assistance (n* = *57 patients)*		
Arrange transportation	41 (CHW)	35 (CHW)
• Fill out form (PT-1) to secure non-emergency medical transportation for patient		
• Call to set up transportation through free services in the city for appointments		
• Work internally within BMC to explore transportation options		
Arrange food assistance		21 (CHW)
• Provide prescriptions for hospital-based food pantry		
• Connect with community-based food pantry		
• Find patients turkeys or grocery store gift cards during the holidays		
Arrange assistance with bill payments		6 (CHW)
• Work with patients to organize and prioritize bill payment for both medical and non-medical bills		
Arrange assistance with utilities		6 (CHW)
• Write letters to utility companies to ensure heat, gas and water provided to patients’ residences		
Provide assistance with housing		8 (CHW)
• Write letters of support for patients with detrimental housing situations- (e.g. mold)		
*COPD management (n* = *57 patients)*		
Education	57 (NP)	36 (NP)
Facilitate pulmonary rehabilitation		5 (NP and CHW)
Arrange non-invasive ventilation/oxygen with DME	11 (NP and CHW)	5 (NP)
Facilitate Scheduling of PFTs		24 (NP and CHW)
*Appointment scheduling (n* = *57 patients)*		
Facilitate scheduling in pulmonary clinic	57 (NP and CHW)	48 (NP and CHW)
Arrange appointments in SUD/MHD clinics		7 (CHW)

Per inclusion criteria, all 57 patients had COPD, ≥ 2 admissions and ≥ 1 unmet SDOH need

35/57 were individuals who currently smoke cigarettes

CHW, community health worker; MHD, mental health disorder; NP, nurse practitioner; NRT, nicotine replacement therapy; PFT, pulmonary function tests; PT-1, Provider Request for Transportation form; SUD, substance use disorder

### Qualitative analysis

Table 5 shows characteristics of participants in qualitative interviews.

**Table 5:** Qualitative interview patient demographics (n = 16)

		Total	% Patients
Gender	Male	11	68.8
Ethnicity	Hispanic origin	2	12.5
Race	Black or African American	3	18.8
	White	11	68.8
	Mixed (African American + American Indian)	1	6.3
Age (years)	< 55	2	12.5
	55–64	9	56.3
	> 65	5	31.3
Insurance status (self-report)	Medicaid (primary or dual-insured)	12	75
	Medicare	1	6.3
	Commercial	1	6.3
	Unknown	2	12.5
Currently smokes cigarettes	Yes	10	62.5
Substance use	Opioids	2	12.5
	Alcohol	6	37.5
	Cocaine	0	0
	Marijuana	2	12.5
	None	7	43.8
	Other	2	12.5
Highest level of education completed	Did not complete high school	8	50.0
	Graduated high school/GED	2	12.5
	Some college, no degree	3	18.8
	Associate or Bachelor's degree	2	12.5
	Graduate or professional degree	1	6.3
Current employment	Full-time	1	6.3
	Part-time	2	12.5
	Unemployed	13	75.0
Housing status	Homeless	2	12.5
Yearly household income (Pre-tax)	$0-$34,999	10	62.5
	$35,000-$74,999	2	12.5
	$75,000-$99,999	1	6.3
	Unsure	3	18.8

GED, General education development

**Fig. 2:**
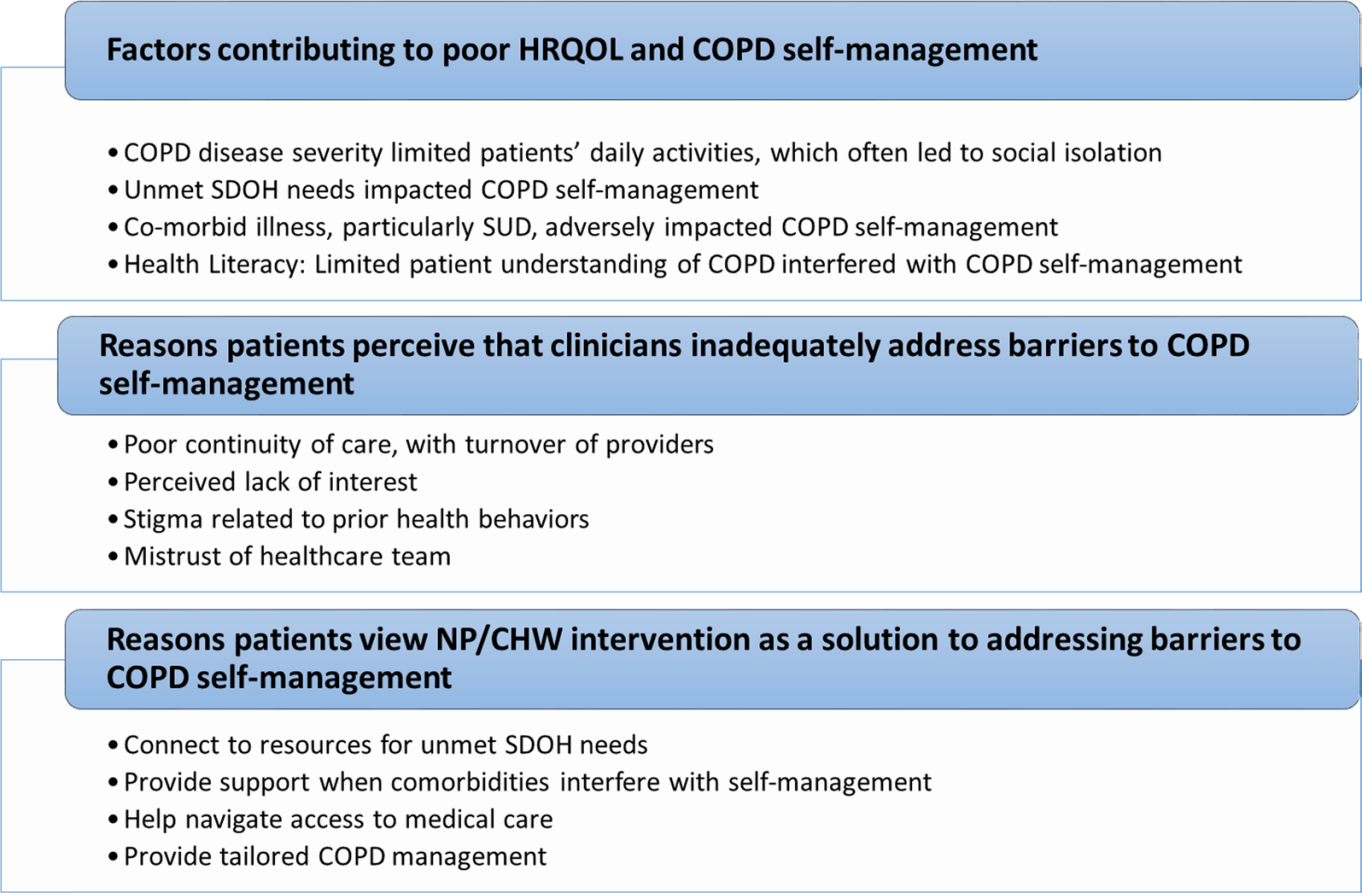
Key themes and subthemes identified from qualitative interviews

Key themes from qualitative interviews included: (1) COPD disease severity, unmet SDOH needs, co-morbid illness, and health literacy led to poor HRQOL and COPD self-management; (2) patients are receptive to receiving help in addressing these factors but perceive clinicians are unable to adequately address them; and (3) patients’ experiences with the CHW were positive (Fig. 2). We elaborate on these themes using key constructs from the Social Contextual Model.

#### Factors contributing to poor HRQOL and COPD self-management

##### COPD disease severity limited patients’ daily activities, which often led to social isolation

How disease severity impacted HRQOL largely depended on patient’s ability to maintain relationships:

I don’t smile. I’m glued to these [oxygen] tubes… I went from someone who went out and people saw at least every day to a ghost. I’m not getting out to socialize and that’s not good for me. (P-4)

When patients received support they perceived better HRQOL.

I have a minister come in at least once a week, which is really nice. I'm hoping if I feel a little better to go to the senior center and maybe take a class or two,…quilting and something else. (P-10)

##### Unmet SDOH needs impacted COPD self-management

Patients cited poor access to transportation and inability to afford medications as barriers to COPD self-management:

Sometimes I can’t pay for my cabs to get to appointments. I got to pay my rent, phone, then dinner. Food is expensive. (P-6)

They ordered me a nice new inhaler. …It would’ve helped me out a lot, but my insurance wouldn’t pay. I don’t have money to pay for it. (P-8)

To improve COPD self-management, patients emphasized how they needed assistance with unmet SDOH needs:

If they get a van to pick me up and make sure I get to my appointments, that’d be great for me. (P-15)

##### Co-morbid illness, particularly SUD, adversely impacted COPD self-management

Patients were aware of and connected how their use of substances made it difficult to self-manage COPD:

It (alcohol) interfered with everything. I’m like, ‘Oh, did I take that [inhaler] today?’ ‘Or was that yesterday I’m thinking of?’ (P-9)

Some patients felt that if they had structure, they would take better care of their health.

I need something to occupy my time, keep me busy, so I won’t fall back to old habits of just sitting and drinking and smoking all day. (P-9)

##### Health literacy: limited patient understanding of COPD interfered with COPD self-management

Patients stated they often had difficulty understanding health-related materials, making it difficult to care for themselves. Navigating the health system was particularly challenging after recent admissions:

I get confused about appointments. I get out of the hospital, I'm confused as hell and everybody hit [me] with their appointments. (P-2)

Patients discussed how being educated about managing COPD would be empowering.

I didn’t know what COPD was. I didn’t never even understand about cigarettes would destroy your body like that. I do not mind making sure I go to a class to make sure I’m using it [inhaler] properly because I might not be getting all the medication that I should be getting in my lungs. …I want to learn everything. If I have to go to these classes, I’m going to go. (P-11)

#### Patients perceive that clinicians inadequately address barriers to COPD self-management

Patients acknowledged they would benefit from support, but did not know how to access it:

There are people [like me] who don’t know how to navigate anything and need help. I just want to make sure everything’s taken care of before I wind up back at home with nothing accomplished. (P-4)

Patients discussed how clinicians’ attempts to address barriers to COPD self-management often failed, stating these reasons:

Poor continuity of care, with turnover of providers: You finally tell [your primary care provider] about everything going on in your life, and then they’re gone. It’s back to square one. I get frustrated about that. (P-1)

Perceived lack of interest: [My] nurse…almost seemed bothered to do anything for me. (P-3)

Stigma related to prior health behaviors: I admit I’m part of the damage after 55 years [of smoking]. When I tell them that, certain doctors are going to have this attitude that ‘He don’t care about himself, why should I care?’ (P-12)

Mistrust of healthcare team: A lot of people [doctors] are all talk and no action. That’s what I find with most of them. ‘Oh, yeah, we’ll get it done,’ and then they don’t. (P-4)

Based on prior experiences, some were reluctant to ask for help.

I don't want nobody to do for me. That's the first thing everybody'd think, because you're in a lower position, …that you're begging. (P-5)

Want to get rid of someone? Ask them for help. Easiest way to get rid of somebody. (P-4)

#### NP/CHW intervention as a solution to addressing barriers to COPD self-management

Almost all patients described their interactions with the NP/CHW as helpful in addressing barriers to COPD self-management, with the following components most helpful:

Connecting to resources for unmet SDOH needs: I have oxygen 24/7. In the summertime, my AC is on all the time. My electric bill is over $5,000. I am very stressed. I don't know how I'm gonna be able to pay that. [The CHW] walked us through the steps and we’re at the point where I'm trying to do a deal with them to start to pay it off. (P-10)

Providing support when comorbidities interfere with self-management: [CHW is] doing all kinds of things that if left up to me, would never get done. I would say F*** it and we’re back to drinking ’til I just died. (P-7)

Navigation to access medical care: I had an appointment April something, and I said, ‘That’s not gonna help me.’ [CHW] got it moved up to like, it’s tomorrow. …Stuff like that really helps. (P-1)

Providing tailored COPD management: I was on [tiotropium], but I was telling them it was getting too hard for me to suck it in. …So they [NP/CHW team] showed me [a different inhaler]. It’s much easier. (P-11)

Some patients, however, had difficulty accepting support and engaging with the NP/CHW team.

I have faith in the system and what people’s trying to do, …but I’m stubborn. If I don’t feel like going, I’m not going. Simple as that. (P-13)

## Discussion

The American Thoracic Society recently called for interventions to address SDOH in COPD populations to reduce disparities, improve care quality, and reduce hospitalizations [22]. In this study, we used mixed methods to inform, pilot test, and evaluate a QI initiative at our safety net hospital for our most vulnerable patients with COPD: individuals with at least two hospitalizations per year and one or more unmet SDOH need. Based on review of EHR data, we learned that almost half of patients with COPD admitted ≥ 2 times per year were Medicaid-insured and 41% were African American, both markers of vulnerability to systemic disadvantage conferred by negative SDOH [23]. Multivariable regression analysis showed being Medicaid-insured and having comorbid cardiac, MHD, and SUD were associated with increased odds of multiple admissions. The majority of patients (74%) with multiple admissions who completed THRIVE had unmet SDOH needs, findings consistent with the growing body of evidence that SDOH drive health outcomes [10, 24]. Patient interviews informed our understanding of the relationship of these factors with poor HRQOL and COPD self-management and gave us insight on how the NP/CHW team performed in addressing these barriers. In short, we found multiple factors contribute to poor HRQOL and COPD self-management among patients with COPD admitted at our safety-net hospital: COPD severity, comorbidities, unmet SDOH needs, and low health literacy. Our discussion focuses on how our findings might influence future interventions both at our own institution and others to promote HRQOL and hospital-free days among frequently admitted patients with COPD.

We found high rates of unmet SDOH needs among frequently admitted patients with COPD. Adverse SDOH increase the risk of poor outcomes after AECOPD [25]. In many clinical contexts, intervening on unmet SDOH needs by connecting individuals with resources can improve health outcomes [26]. Few interventions focus on how screening and addressing SDOH might improve COPD outcomes. Our study suggests that patients hospitalized at our safety-net hospital are receptive to such interventions, as has been previously shown in our primary care clinics [16].

Our QI initiative pairing a disease-specific trained NP to deliver evidence-based COPD care and a CHW to address unmet SDOH needs demonstrated that the majority of patients engaged with the CHW post-discharge and received assistance with unmet SDOH needs. In qualitative analyses, patients participating in the NP/CHW intervention described how COPD severity, comorbidities, unmet SDOH needs, and poor health literacy influenced HRQOL and ability to navigate their healthcare. While patients hesitated asking clinicians for help in addressing these factors, they were receptive to CHW-facilitated tailored assistance.

Patients identified components as particularly helpful: (1) connecting to resources for unmet SDOH needs; (2) providing support for social isolation; (3) navigating to access medical care; and (4) providing tailored COPD management. Among patients with COPD, perceived social support is associated with improved self-efficacy [27, 28] Studies have evaluated the effectiveness of CHWs in improving breast cancer screening and health outcomes including cardiac disease [29–32]. A systematic review on CHW interventions demonstrated improvement in aspects of asthma-related disease burden; no studies have reported on COPD-specific outcomes [33].

A strength of this work is our mixed-methods approach to inform development and evaluation of the QI initiative, including analysis of sociodemographic and comorbidity data from the EHR, screening for SDOH needs using a standardized instrument (THRIVE screener) [16], review of NP/CHW logs, and qualitative interviews. Another strength was the focus on the understudied population of COPD patients frequently admitted to a safety-net hospital. The major limitations of our study include small sample size and single site which limits generalizability. Further, findings are from participants who volunteered and may not reflect perspectives from all individuals. This preliminary evaluation focused on assessing feasibility and acceptability of the QI initiative, but was not designed to comment on clinical effectiveness.

## Conclusions

Mortality in individuals with COPD has decreased faster in individuals with the highest compared with the lowest SES [34]. A meta-analysis showed that COPD discharge care bundles focused on medical management lead to fewer readmissions but no improvements in mortality and HRQOL [35]. Several SES domains are risk factors for COPD readmission or death [36], yet discharge bundles rarely address factors that low SES patients face. In this study we show it is essential for COPD discharge bundles in hospitals serving marginalized populations to address how SDOH and comorbidities influence COPD self-management. Our longitudinal NP/CHW intervention appears to be a feasible and acceptable strategy to intervene on these factors and is responsive to the American Thoracic Society’s call for interventions to address SDOH needs for patients with COPD. Future work will assess effectiveness of this intervention at reducing hospitalizations.

## Supplementary Information

**Additional file 1:** THRIVE screening tool.

## Data Availability

The data that support the findings of this study are available from the corresponding author upon request.
