# Supplementary material for: A mixed methods study to inform and evaluate a longitudinal nurse practitioner/community health worker intervention to address social determinants of health and chronic obstructive pulmonary disease self-management
Source: BMC Pulm Med. 2022 Mar 1;22:74. doi: 10.1186/s12890-022-01863-w (PMC8889692; doi:10.1186/s12890-022-01863-w)
Supplement: Supplementary file 1 — Additional file 1: THRIVE screening tool. [file 12890_2022_1863_MOESM1_ESM.docx]

**Additional file 1**

**Patient Interview Guide**

**Do you currently smoke cigarettes? (Please choose only one)**

- Every day
- Some days
- Not at all
- Refused

**Do you feel stress, tense, restless, nervous, or anxious, or unable to sleep at night because your mind is troubled all the time-these days?**

- Not at all
- Only a little
- To some extent
- Rather much
- Very much
- Patient refused

**In a typical week, how many times do you talk on the phone with family, friends, or neighbors?**

- Never
- Once a week
- Twice a week
- Three times per week
- More than three times per week
- Patient refused

**How often do you get together with friends or relatives?**

- Never
- Once a week
- Twice a week
- Three times per week
- More than three times per week
- Patient refused

**How often do you attend church or religious services?**

- Never
- 1 to 4 times per year
- More than 4 times per year
- Patient refused

**Do you belong to any clubs or organizations such as church groups, unions, fraternal or athletic groups, or school groups?**

- Yes
- No
- Patient refused

**How often do you attend meetings of the clubs or organizations you belong to?**

- Never
- 1 to 4 times per year
- More than 4 times per year
- Patient refused

We are trying to learn how we can best address any challenges patients with COPD, such as yourself, may have in getting the health care they need, particularly after leaving the hospital. There are two ways we are thinking about doing this and are interested in what you think.

- Would you be interested in receiving information sheets at discharge and again in the mail about services and resources that might be helpful to you (e.g. education on COPD and/or tobacco meds, social services)?
- Yes
- No
- Unsure
- Patient refused
- Would you be interested in having a trusted member from the community (called a community health worker) meet you while you are in the hospital and check in again with you after discharge for one to three months to help you get the services and resources you may need?
- Yes
- No
- Unsure
- Patient refused

**Participant Number**
